# Recombinant α-Toxin BmK-M9 Inhibits Breast Cancer Progression by Regulating β-Catenin In Vivo

**DOI:** 10.1007/s12013-025-01711-8

**Published:** 2025-03-13

**Authors:** Wenlin Chen, Zhuocen Cha, Saijun Huang, Ruimin Liu, Jiayi Chen, Peter Muiruri Kamau, Xingjia Lu, Bowen Li, Dequan Liu

**Affiliations:** 1 Department of Breast Surgery, The Third Affiliated Hospital of Kunming Medical University, Peking University Cancer Hospital Yunnan, Yunnan Cancer Hospital, Kunming, Yunnan China; 2 Oncology department, Guizhou Hospital of the First Affiliated Hospital, Sun Yat-sen University, Guiyang, Guizhou China; 3 Maternal and Child Health Hospital of Changsha County, Changsha, Hunan China; 4 Key Laboratory of Animal Models and Human Disease Mechanisms of Chinese Academy of Sciences/Key Laboratory of Bioactive Peptides of Yunnan Province/National & Local Joint Engineering Center of Natural Bioactive Peptides, Kunming Institute of Zoology, Chinese Academy of Sciences, Kunming, Yunnan China; 5 National Resource Center for Non-Human Primates, Kunming Primate Research Center/National Research Facility for Phenotypic & Genetic Analysis of Model Animals (Primate Facility), Kunming Institute of Zoology, Chinese Academy of Sciences, Kunming, Yunnan China; 6 Department of Breast Surgery, The First Affiliated Hospital of Kunming Medical University, Kunming, Yunnan China

**Keywords:** β-catenin, Breast cancer, BmK-M9, Invasiveness, Metastasis, Proliferation

## Abstract

Screening bioactive compounds from natural sources, including animals and plants, is a valuable strategy for identifying novel anti-tumor agents. α-Toxin BmK-M9, a key component of scorpion venom, has received limited attention regarding its potential anti-cancer effects and underlying mechanisms in breast cancer. This study investigates the effects and mechanisms of BmK-M9 in breast cancer using in vitro experiments and a nude mouse model. mRNA sequencing was performed to identify affected signaling pathways, while Western blotting and immunohistochemistry were utilized to analyze the Wnt/β-catenin signaling pathway. The results demonstrated that BmK-M9 significantly inhibited breast cancer cell invasion and migration in vitro and suppressed tumor growth in vivo. Transcriptomic analysis revealed that BmK-M9 influenced cellular processes related to proliferation, apoptosis, motility, and metabolism. Furthermore, BmK-M9 markedly downregulated β-catenin expression in the Wnt/β-catenin pathway. These findings suggest that BmK-M9 exerts anti-tumor effects in breast cancer by modulating Wnt/β-catenin signaling, highlighting its potential as a promising therapeutic candidate.

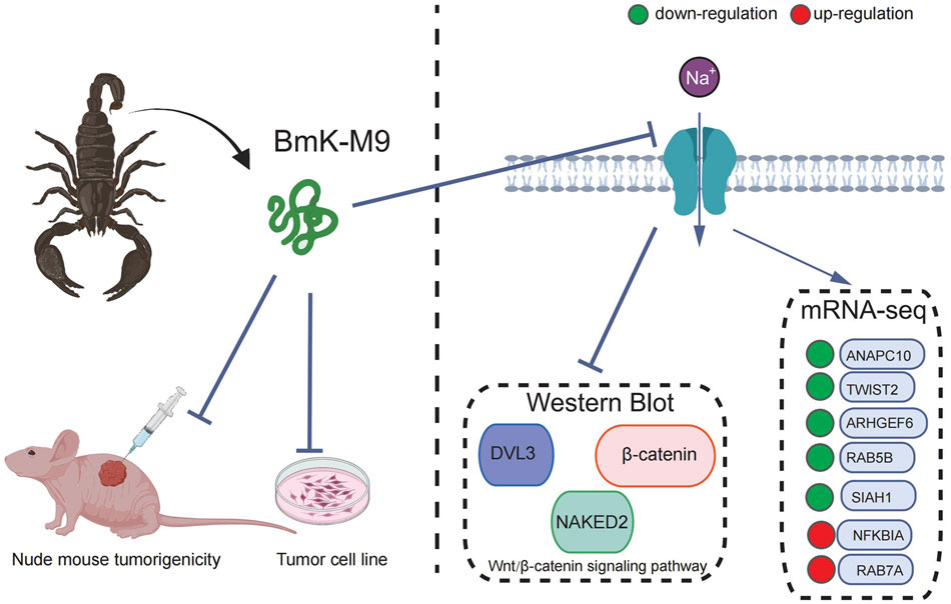

## Introduction

Breast cancer remains a major global health issue and is the most common cancer among women worldwide. According to the latest data from GLOBOCAN, there were 2.297 million new cases of breast cancer and 666,000 deaths globally in 2022. Breast cancer is the leading cancer among women in terms of both incidence and mortality, accounting for 23.8% of the total incidence and 15.4% of the total mortality of cancers in women worldwide [1].

Research has demonstrated the effectiveness of various natural compounds in the prevention and treatment of cancer, with fewer side effects compared to traditional radiotherapy and chemotherapy [2, 3]. Screening potential therapeutic drugs from bioactive products derived from animals and plants is a valuable approach [4]. Venom obtained from scorpions, snakes, and spiders has been identified as a promising source for developing anticancer treatments [5–8]. Notably, several components of scorpion venom have shown potential anticancer activity, including against breast cancer [9–11]. α-Toxin BmK-M9 is a component of *Buthus martensii* Karsch scorpion venom, known primarily for its inhibitory effect on sodium channels’ rapid repolarization [12]. However, the effects of BmK-M9 on breast cancer cells and the specific mechanisms involved remain poorly understood.

The occurrence and progression of breast cancer are influenced by multiple signaling pathways, including the Wnt/β-catenin pathway, JAK-STAT signals, Notch, TGF-β, Hedgehog, Hippo YAP PI3K/AKT/mTOR, and Epidermal Growth Factor Receptor, among others [11]. Abnormal activation of the Wnt/β-catenin pathway has been implicated in the development of breast cancer [3, 13, 14]. A growing body of evidence suggests that the Wnt/β-catenin pathway is associated with various aspects of cancer, including proliferation [15], metastasis [16], stemness maintenance [17], therapeutic resistance [18], phenotype regulation [19], and immune microenvironment modulation [20–22]. Consequently, targeting and inhibiting the Wnt/β-catenin pathway holds great potential for breast cancer treatment. However, there are currently no approved inhibitors specifically targeting the Wnt/β-catenin pathway for breast cancer therapy.

In our study, we aim to investigate the anticancer effects of BmK-M9 on breast cancer and explore its underlying mechanisms. Through in vitro and in vivo experiments, we plan to evaluate the biological impact of BmK-M9 on breast cancer cells, particularly its inhibitory effects on cell proliferation, invasion, metastasis, and tumor growth. Furthermore, this study will explore the potential regulatory role of BmK-M9 in the Wnt/β-catenin signaling pathway, with the goal of providing a new therapeutic strategy for breast cancer treatment.

## Materials and Methods

### Expression and Purification of BmK-M9

The nucleotide sequence of BmK-M9 is 5′GGTACCGAAAATCTGTACTTCCAAGGCGTGCGTGATGCGTATATTGCGAAACCGGAAAACTGTGTCTACCATTGTGCGACGAACGAAGGCTGTAACAAACTGTGCACCGATAATGGCGCGGAAAGCGGTTATTGTCAGTGGGGCGGTCGTTACGGCAACGCGTGTTGGTGTATCAAACTGCCGGACCGTGTGCCGATTCGTGTGCCGGGTAAATGCCATTAAAAGCTT-3′. The synthetic BmK-M9 sequence was inserted into the prokaryotic expression vector pET-32a(+) (Novagen). Recombinant *E. coli* X1X5X4BH-28a-BL21(DE)3 was used for protein expression. Protein expression was induced, and the resulting protein was purified using a nickel column and high-performance liquid chromatography (HPLC). The fusion tag of the protein was subsequently removed using the rTEV enzyme to obtain a highly purified form of BmK-M9.

### Cell Culture

HEK293T, MDA-MB-231, SUM149PT, and MCF7 cell lines were procured from the Kunming Institute of Animal Science, Chinese Academy of Sciences. HEK293T and MCF-7 cells were cultured in DMEM (Corning) supplemented with 10% fetal bovine serum and 1% penicillin mixture (Solarbio). SUM149PT cells were cultured in DMEM/F12 (1:1) medium (Corning) supplemented with 10% fetal bovine serum and 1% penicillin mixture (Solarbio). MDA-MB-231 cells were cultured in L-15 medium (Gibco) supplemented with 10% fetal bovine serum and 1% penicillin mixture (Solarbio). All cell lines were maintained in a humidified incubator at 37 °C with 5% CO_2_. The cells were kept within 20 passages to ensure consistency and reliability of results.

### Verification of Biological Activity of BmK-M9

To verify the biological activity of BmK-M9, electrophysiological experiments were conducted using HEK293T cells overexpressing Na_V_1.5. The Via Fect transfection reagent (Promega E4981) was used for cell transfection. The extracellular solution was prepared with the following composition: NaCl 140 mM, KCl 4 mM, MgCl_2_ 1 mM, CaCl_2_ 2 mM, glucose 11.1 mM, and HEPSE 10 mM, adjusted to pH 7.4. The pipette solution contained KCl 130 mM, NaCl 15 mM, CaCl_2_ 0.37 mM, MgCl_2_ 1 mM, MgATP 1 mM, EGTA 1 mM, and heparin 10 mM, adjusted to pH 7.2.

### Whole-Cell Membrane Clamp Experiments

These experiments were performed at room temperature (20–22 °C). The experimental setup included a puller (Sutter P97) for the fabrication of thin-walled borosilicate glass electrodes (WPI). The electrodes were polished using a Narishige MF-830 polisher. A microscope (Olympus IX73) was used for visualization. The glass electrodes were connected to a recording probe (Headstage) and then to an amplifier/digital-to-analog converter (HEKA EPC 10 USB). Using a micromanipulator (Sutter MP-285), the glass electrode was carefully positioned near the Na_V_1.5-overexpressing HEK293T cell. A short, forceful negative pressure was applied to the sealed cell, causing the clamped membrane to rupture. The entire cell was slowly lifted by micromanipulation, and the recording mode was switched to whole-cell configuration. The HEK293T cells were placed in the RSC-200 (BioLogic) Rapid Perfusion Dosing System, and 1 μM BmK-M9 was added through the bath solution channel connected to the 8-link tubes. Current traces of the HEK293T cells were recorded before and after the addition of BmK-M9 using the whole-cell recording mode. Sodium channel recordings were obtained with a clamping voltage of −100 mV for 50 ms, a test voltage of −10 mV for 100 ms, and a clamping voltage of −100 mV for 50 ms.

### Invasion Experiment

In this experiment, three breast cancer cell lines, namely MDA-MB-231, SUM149PT, and MCF-7, were utilized to investigate their invasion capabilities. To prepare the stromal gel, a 1:5 mixture of BD:356234 with serum-free medium was incubated in the upper chamber at 37 °C for 30 min. Cells were then detached using trypsin and resuspended in serum-free medium to achieve a cell density of 5 × 10^5^ cells/ml. For the experimental group, 1 μM BmK-M9 was added. In the lower chamber, 900 μl of complete medium was added, and the entire setup was then incubated at 37 °C with 5% CO_2_ for 24 h. Subsequently, the chambers were carefully removed, and the excess matrix gel was gently wiped off using cotton swabs. The lower membranes were fixed and stained with crystal violet, followed by decolorization. Finally, the membranes were washed, dried, placed under a microscope, and observed for cell invasion. Photographic documentation was performed for record-keeping. Each treatment concentration was tested 3–6 times, and the results were compiled as the average number of replicates.

### MTT Assay

For the MTT assay, three breast cancer cell lines, MDA-MB-231, SUM149PT, and MCF-7, were employed. The cells were trypsinized using Corning trypsin and adjusted to a density of 5 × 10^3^ cells per well. Subsequently, the cells were evenly distributed in a 96-well plate and starved in serum-free medium for 16 h. Each cell line was divided into the following groups: a control group treated with phosphate-buffered saline (PBS), a BmK-M9-treated group with a concentration gradient of 2, 1, 0.5, 0.25, 0.125, and 0.0625 µM, a positive control group treated with YL109 (from TargetMol), and a negative control group treated with FGF2 (from MedChemExpress). Each group consisted of three wells as duplicates. After 24 h of drug treatment, 10 μl of MTT (from Bioshap) was added to each well. Following a 2 h incubation period, the 96-well plate was removed, and the absorbance at 490 nm was measured using a microplate reader. The effects of BmK-M9 on the proliferation of breast cancer cells were compared and analyzed.

### Cell Migration Assays

Scratch wound assays were conducted in 6-well plates. The cell lines were seeded at a density of 1×10^6^ cells/well and cultured until reaching confluence. Subsequently, the media was replaced with media containing BmK-M9 (1 μM) or media without BmK-M9. A scratch wound was created using a pipette tip. Photographic documentation was performed at 0 and 48 h to capture the changes in the scratches. The images were processed using ImageJ software, and the area within the scratches was measured at different time points. Each experiment was independently repeated at least three times.

### Flow Cytometry Analysis

Flow cytometry analysis was performed using the Annexin V-FITC/PI apoptosis detection kit (Meilunbio MA0220-2) to assess the apoptosis rate of breast cancer cells treated with BmK-M9. PI staining was used to investigate the cell cycle phase. After 48 h of co-incubation with BmK-M9, breast cancer cells were treated according to the manufacturer’s instructions. Fluorescence signals were detected using a flow cytometer (Fortessa, USA). The obtained data were analyzed using Flowjo software, and statistical analysis of apoptosis rate and cell cycle distribution was conducted using GraphPad software.

### Animal Ethics Statement and Experimental Procedures

All animal experiments conducted in this study were approved by the Ethical Review Board of Kunming Institute of Zoology, Chinese Academy of Sciences. The approval reference number is IACUC-RE-2022-07-003, and the experiments followed the National Institutes of Health Guidelines for Animal Experimentation. Female Balb/c nude mice (4–6 weeks old) were obtained from Shanghai Southern Biotechnology Co. MDA-MB-231 cells were harvested, digested, and centrifuged. The cells were then resuspended in a mixture of PBS and Matrigel (BD, 354230) gel at a 1:1 ratio, which was kept on ice to prevent gel solidification. Subsequently, the cells (approximately 3×10^6^ in volume) were injected into the mammary fat pad of the nude mice using a 100 μl homogenized system. When the tumor volume reached approximately 0.1 cm^3^, drug administration began. The drugs were administered intraperitoneally for 5 consecutive days per week with a 2-day interval. The administration time is 9:00 a.m., and the injection time for each mouse is between 20–30 seconds to ensure full absorption of the drug. The experimental groups are divided as follows: control group (normal saline), positive control group (ranolazine 50 mg/kg), and BmK-M9 treatment group (BmK-M9 1 mg/kg). There are 5 mice in each group, and the injection volume is 1.5 ml. After one month of continuous administration, the mice are euthanized by cervical dislocation under anesthesia, and the tumors are carefully and completely excised. Semi-quantitative immunofluorescence analysis was performed to evaluate the differences in Ki67 content among the tumor samples from the different groups.

### Sequencing Process

MDA-MB-231 cells were cultured in a 6-well plate. After the cells were full-well, the old medium was removed, and MDA-MB-231 cells were treated with 1 μM BmK-M9 (dissolve in medium) for 48 h, there were three biological replicates in the experimental group and the control group. After treatment, the medium was removed, and cell samples were collected using 1 ml of TRIZOL per well. After treatment, the medium was removed, and cell samples were collected using 2 ml of TRIZOL per well. Total RNA was extracted using MJzol Animal RNA Isolation Kit (Majorivd) and according to the standard operating procedures provided by the manufacturer. Purification was performed using the RNAClean XP Kit (Beckman Coulter) and RNase-Free DNase Set (QIAGEN). RNA integrity was detected by Agilent 2100 Bioanalyzer/Agilent 4200 TapeStation (Agilent technologies). The total amount and purity of RNA were determined by Qubit 2.0 fluorescence quantifier (Thermo Fisher Scientific) and NanoDrop ND-2000 spectrophotometer (Thermo Fisher Scientific). Library preparation according to the standard instructions for the VAHTS Universal V6 RNA-seq Library Prep Kit for Illumina (Illumina). The concentration and size distribution of the cDNA library were assessed using the Agilent 4200 bioanalyzer before sequencing on an Illumina novaseq6000 platform. The high-throughput sequencing protocol was strictly followed as per the manufacturer’s instructions (Illumina). The raw reads obtained from sequencing were filtered using Seqtk, the main steps are as follows: 1. Remove the linker sequence contained in reads; 2. The base error rate is less than 0.01 when Q is less than 20 at the 3′ end. Among them, Q = −10 logerror 3. Reads with length less than 25; 4. Ribosome RNA reads of the species. And then mapped to the genome using Hisat2 (version: 2.0.4), the genomic version is GRCM38. The gene fragments were quantified using stringtie (v1.3.3b), and subsequently normalized using TMM (trimmed mean of M values) normalization, the FPKM value of each gene is calculated by Perl script. Differential gene expression analysis was performed using edgeR software, where genes with a False Discovery Rate (FDR) value below the threshold of Q < 0.05 and a fold-change greater than 2 were considered significant differentially expressed genes (DEGs). The differential genes were analyzed by METAscape for function enrichment, the protein-protein interaction network was analyzed by STRING, and the data were displayed by R.

### Molecular Level Validation by Western Blot

MDA-MB-231 breast cancer cells were treated with BmK-M9 (1 μM) and incubated at 37 °C in a humidified atmosphere containing 5% CO_2_. Protein samples were collected at 3, 6, 12, 24, and 48 h. To prepare cell lysates, protease and phosphatase inhibitors (MedChemExpress) were added to the lysis buffer (Sigma-Aldrich), and the mixture was applied to different cell groups, followed by incubation on ice for 30 min. Proteins from myocardial tissue lysates were separated using Omni-PAGE™ Precast Gel Hepes (4–20%, 15 wells) (Yame, LK213) and transferred onto PVDF membranes. The membranes were blocked with 5% BSA (Albumin Fraction V, Biofroxx, 4240GR500) for 2 h. Subsequently, they were incubated overnight at 4 °C with primary antibodies, including P-LRP6, DVL3, DVL2, AXIN1, and NAKED2 (1:1000; Wnt Signaling Antibodies Sampler Kit, cat. no. 2915; Cell Signaling Technology, Inc.), β-catenin (1:1000; cat. no. AF6266; Affinity, Inc.), Cyclin D1 (1:1000; cat. no. AF0931; Affinity, Inc.), GAPDH (1:5000; cat. no. AF7021; Affinity, Inc.), and WNT5A (1:1000; cat. no. AF7021; Affinity, Inc.). Following incubation with a secondary antibody (Affinity), protein bands were visualized using a fully automated chemiluminescence/fluorescence imaging system (Tanon 5200 Multi). The grayscale intensity of the bands was quantified using ImageJ software for data analysis.

### Immunohistochemistry (IHC)

The expression of β-catenin was evaluated through immunohistochemistry (IHC). Mouse tumor tissues were fixed with 4% paraformaldehyde, and after undergoing dehydration, transparency, paraffin embedding, sectioning, and mounting procedures, they were sliced into sections. The sections were deparaffinized in xylene, rehydrated through an ethanol series, and subjected to antigen retrieval using EDTA antigen extraction buffer with microwave boiling. Endogenous peroxidase activity was blocked by incubation with 3% hydrogen peroxide at room temperature for 10 min. Subsequently, the sections were incubated overnight at 4 °C with an anti-β-catenin antibody. On the following day, the sections were incubated with secondary antibodies for 30 min at 37 °C. After thorough washing, they were stained using a diaminobenzidine peroxidase substrate kit (Impact DAB) and counterstained with hematoxylin. The slides were then dehydrated, sealed with resin, and examined under a microscope. The protein levels were analyzed using ImageJ software.

### Statistical Analysis

Quantitative data are expressed as mean ± standard deviation (SD). Multiple group comparisons were statistically analyzed using one-way or two-way analysis of variance (ANOVA). Comparisons between two groups and among multiple groups were performed using Student’s t-test and one-way ANOVA, respectively. Statistical analysis was performed using GraphPad Prism 9.0 software (GraphPad Software, Inc., La Jolla, CA, USA). Statistically significant differences were denoted as ***p < 0.001, **p < 0.01, and *p < 0.05.

## Results

### Verification of BmK-M9 Purification and its Invasion Inhibition in Breast Cancer Cells

**Fig. 1:**
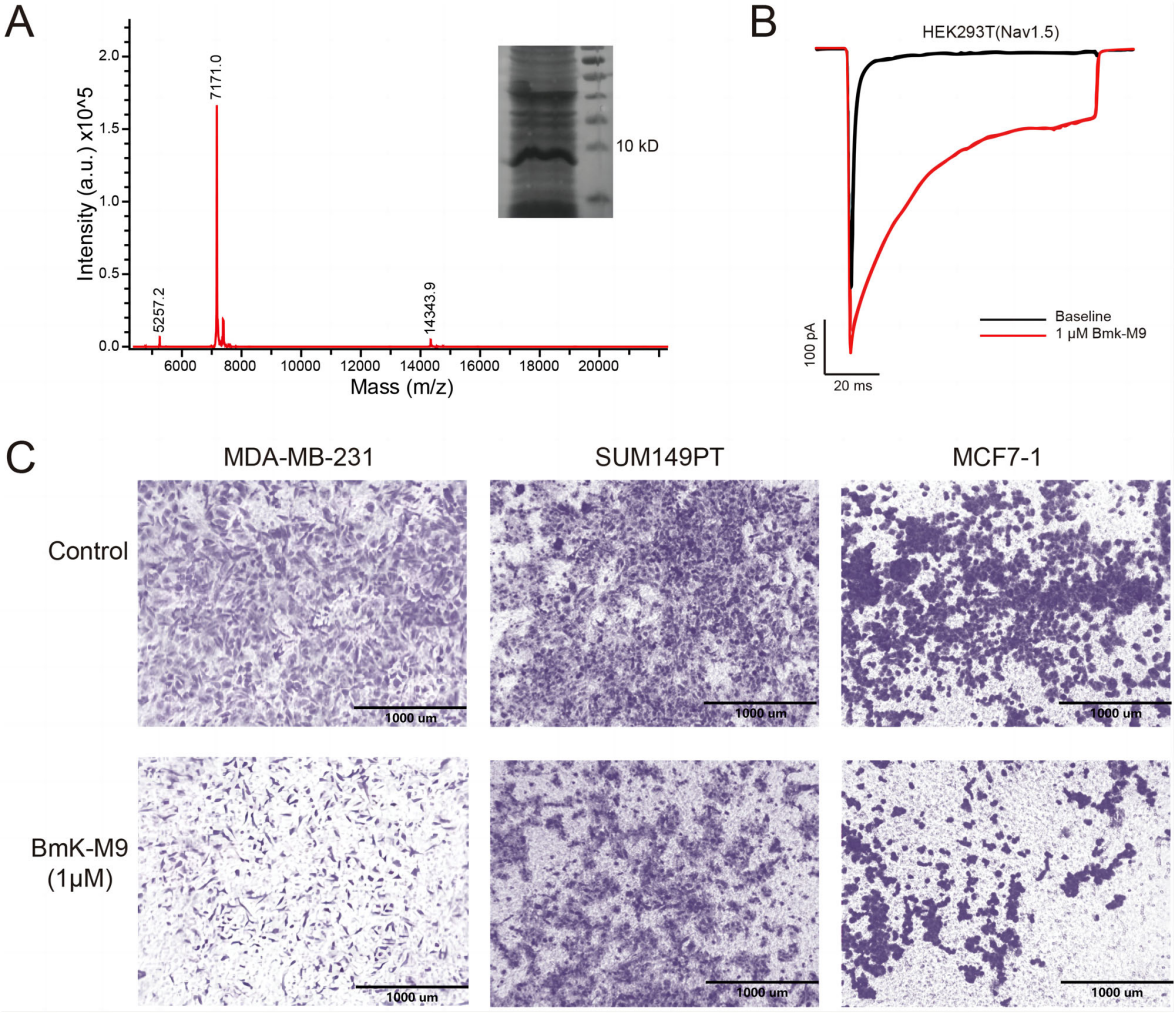
Identification of bioactivity of recombinant BmK-M9 protein and the effect of BmK-M9 on invasion of breast cancer cells. **A** SDS-PAGE and MALDI-TOF-MS for the molecular weight identification of recombinant BmK-M9 protein. **B** Electrophysiological experiments conducted in HEK293T cell lines overexpressing Na_V_1.5, treated with BmK-M9 or intracellular fluid. **C** Stromal gel invasion assay performed on MDA-MB-321, SUM149PT, and MCF-7 cells treated with BmK-M9 or PBS, respectively

To obtain bioactive BmK-M9 protein, an expression vector was constructed, and the fusion protein was induced as described [23]. SDS-PAGE analysis confirmed the correct expression of BmK-M9 in *E. coli* (Fig. 1A). The elution peaks of BmK-M9 protein were determined to be 7171.0 Da using MALDI-TOF-MS spectroscopy (Fig. 1A, Line graph), indicating successful synthesis of BmK-M9. BmK AGAP, an analogue of BmK-M9 derived from the Scorpion *Buthus martensii* Karsch, is known for its specific inhibition of Na^+^ channels [24] and exhibits a corresponding effect on Na^+^ channels. Therefore, the patch clamp technique was employed to evaluate the impact of synthetic BmK-M9 on HEK293T cells overexpressing Na_V_1.5 in the whole-cell recording mode. The results demonstrated that HEK293T cells transfected with Na_V_1.5 exhibited sodium currents, and treatment with 1 μM BmK-M9 significantly inhibited rapid sodium channel repolarization (Fig. 1B). These findings indicate that the purified BmK-M9 protein possesses similar biological activity to its analogues. Previous studies have demonstrated the inhibitory effect of BmK AGAP on breast cancer [25]. To investigate the impact of BmK-M9 on breast cancer cell invasion, three different breast cancer cell lines, namely MDA-MB-231, SUM149PT, and MCF-7, were treated with 1 μM BmK-M9 or an equal volume of PBS in a cell invasion assay. The BmK-M9-treated group displayed significantly reduced cancer cell invasion compared to the control group across all three cell lines, suggesting a prominent inhibitory effect of BmK-M9 on breast cancer cell invasion (Fig. 1C).

### BmK-M9 Inhibits Migration and Viability in Breast Cancer Cells

**Fig. 2:**
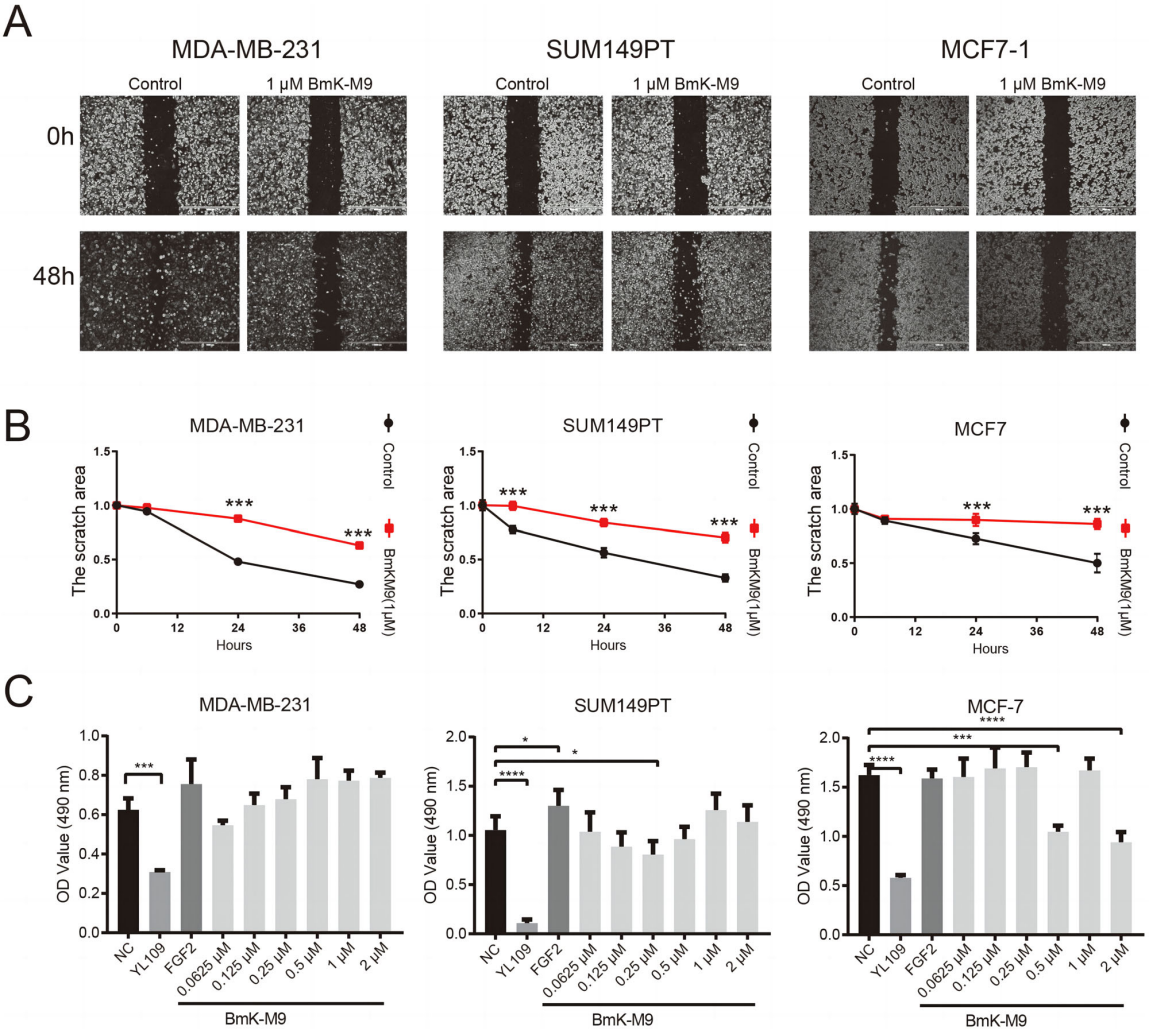
BmK-M9 inhibits the migration and viability of breast cancer cells. **A** Scratch healing assay showing the effect of BmK-M9 treatment on the scratch closure of three different subtypes of breast cancer cell lines at 0 h and 48 h. **B** Statistical analysis of the wound healing assay data for three groups of cells at 0 h, 6 h, 24 h, and 48 h (*n* = 3, one-way ANOVA *: 0.01 < *p* < 0.05; **: 0.001 < *p* < 0.01; ***: 0.0001 < *p* < 0.001; ****: *p* < 0.0001). **C** MTT assay measuring the viability of three different subtypes of breast cancer cell lines treated with different concentrations of BmK-M9 for 24 h (*n* = 3, one-way ANOVA *: 0.01 < *p* < 0.05; **: 0.001 < *p* < 0.01; ***: 0.0001 < *p* < 0.001; ****: *p* < 0.0001)

The scratch healing assay was employed to investigate the impact of BmK-M9 on breast cancer migration. Scratches were created on breast cancer cell monolayers (MDA-MB-231, SUM149PT, and MCF7 cells) and subsequently treated with 1 μM BmK-M9. The width of the scratches was measured at 6 h, 24 h, and 48 h. Our results demonstrated that after 48 h of treatment, the width of the scratches in the breast cancer cells (MDA-MB-231, SUM149PT, and MCF7 cells) treated with 1 μM BmK-M9 was wider compared to the control group (Fig. 2A). Furthermore, the healing speed of the scratches in MDA-MB-231, SUM149PT, and MCF7 cells treated with BmK-M9 was significantly slower than that of the control group. Particularly noteworthy was the significant effect observed as early as 6 h after treatment in SUM149PT cells (Fig. 2B). These findings indicate that BmK-M9 significantly inhibits the migratory ability of breast cancer cells. Subsequently, the aforementioned three breast cancer cell lines were treated with various concentrations of BmK-M9, and the effect of BmK-M9 on cell viability was assessed using the MTT assay. Our experimental results revealed that BmK-M9 had no significant effect on the viability of MDA-MB-231 cells. However, at a concentration of 0.25 μM, BmK-M9 significantly inhibited the viability of SUM149PT cells. Similarly, at concentrations of 0.5 and 2 μM, BmK-M9 significantly inhibited the viability of MCF7 cells (Fig. 2C). These findings indicate that BmK-M9 exerts a suppressive effect on breast cancer cell viability, to some extent.

### BmK-M9 Promotes Apoptosis in MDA-MB-231

**Fig. 3:**
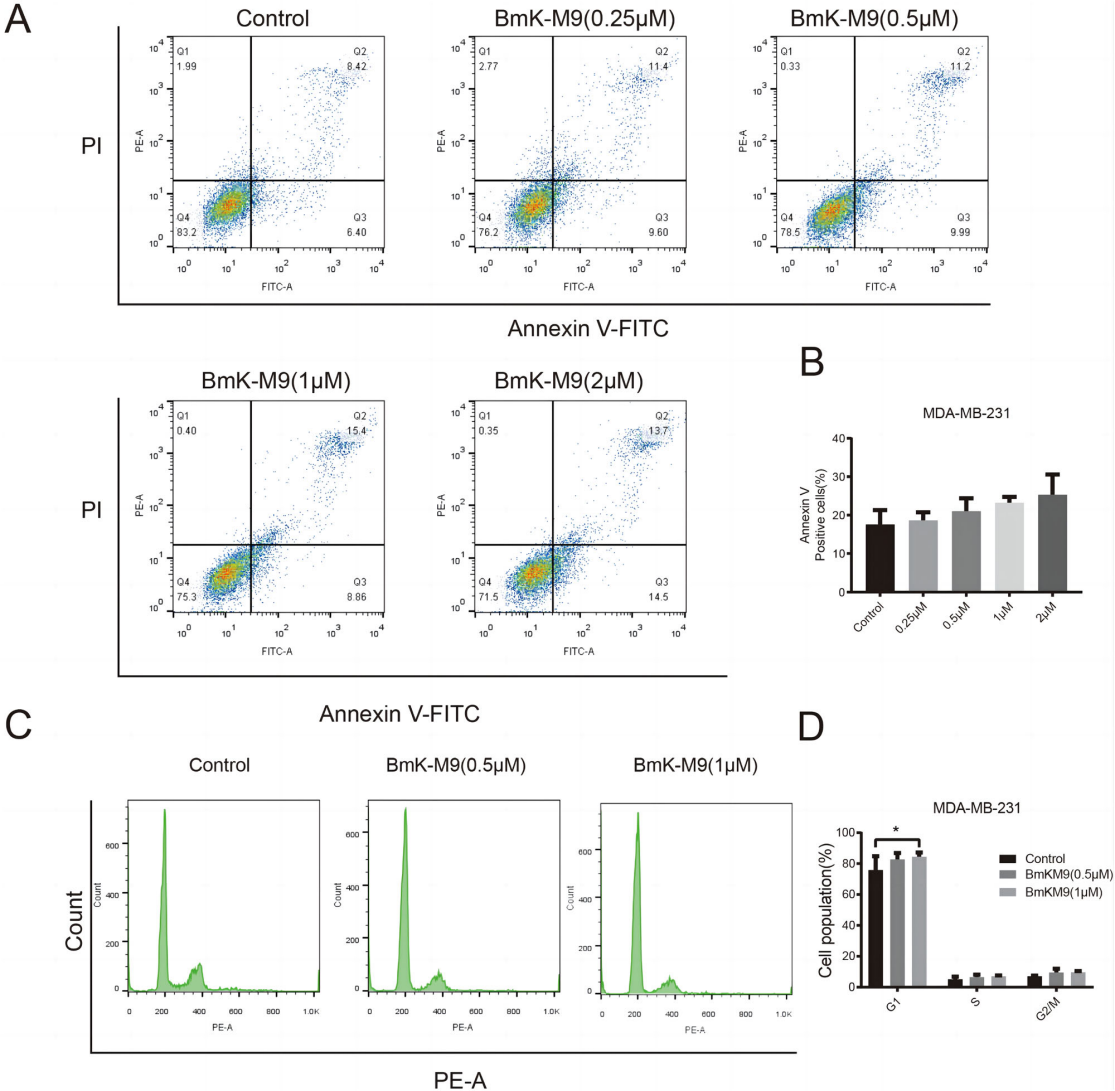
The effect of BmK-M9 on apoptosis in MDA-MB-231 cells. **A** and **B** Analysis of apoptosis in MDA-MB-231 cells treated with BmK-M9 for 24 h using flow cytometry. **C** and **D** Flow cytometry analysis of the cell cycle revealed that BmK-M9 induced cell cycle arrest at the G1 phase in MDA-MB-231 cells (*n* = 3, one-way ANOVA *: 0.01 < *p* < 0.05; **: 0.001 < *p* < 0.01; ***: 0.0001 < *p <* 0.001; ****: *p* < 0.0001)

Apoptosis, also known as programmed cell death, is a natural process involved in the removal of senescent cells from the body [26]. Most anticancer therapies exert their effects by inducing apoptosis and activating relevant cell death pathways [27]. To investigate the impact of BmK-M9 on apoptosis in breast cancer cells, MDA-MB-231 cells were treated with varying concentrations of BmK-M9 (0.25, 0.5, 1, and 2 μM). Our findings revealed that BmK-M9 had an effect on the necrosis of breast cancer cells, although its impact was more pronounced on late-stage apoptosis in MDA-MB-231 cells (Fig. 3A). Additionally, the overall tendency of BmK-M9 to promote apoptosis in breast cancer cells was dose-dependent, although statistical significance was not reached (Fig. 3B). Flow cytometry cell cycle analysis demonstrated that MDA-MB-231 cells treated with BmK-M9 tended to accumulate in the G1 phase, with a stronger effect observed at higher concentrations (Fig. 3C, D).

### BmK-M9 Suppresses Breast Tumor Growth and Reduces Ki67 Expression in Nude Mice

**Fig. 4:**
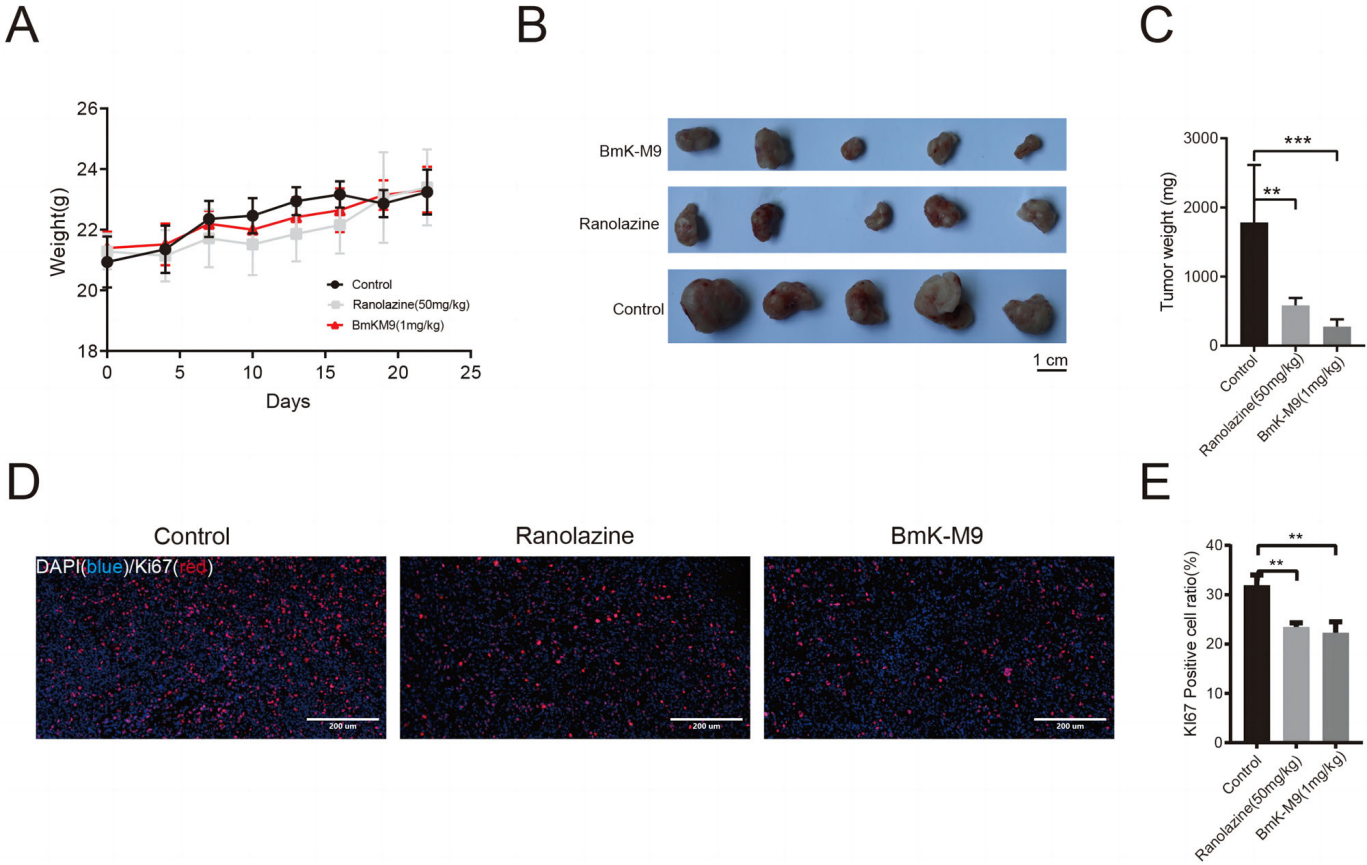
BmK-M9 inhibits the growth of breast cancer in vivo. BALB/C nude mice were subcutaneously inoculated with MBA-MB-231 cells, and once visible tumors appeared, they were injected with either the vector or BmK-M9 into the peritoneum once a day. **A** Monitoring of body weight in mice. **B** Images of excised tumors from the different treatment groups after 20 days of tumor implantation. **C** Quantitative analysis of excised tumor weight. Mice were sacrificed after 20 days, and the tumors were removed and weighed. **D** Immunofluorescence analysis of Ki67-positive (Ki67^+^) cells in each group. Blue represents DAPI staining, and red indicates Ki67^+^ cells. **E** Percentage of Ki67^+^ cells in each group (**p < 0.01, *n* = 5)

To further investigate the potential anti-cancer effect of BmK-M9 in vivo, an experiment was conducted using nude mice as a model for transplanted breast cancer. Female nude mice were injected with a mixture of 100 μL Matrigel (BD, 354230) and MDA-MB-231 cells (1:1 ratio) into the breast fat pad. When the tumor volume reached approximately 0.1 cm, mice were treated with intraperitoneal injections of 1 mg/kg BmK-M9 (or 50 mg/kg Ranolazine) five days a week, with a two-day interval. The control group received an equivalent volume of normal saline. The results showed that BmK-M9 did not have a significant effect on the body weight of mice compared to the control group after tumor implantation (Fig. 4A). However, BmK-M9 significantly suppressed tumor growth compared to the control group (Fig. 4B, C). Notably, its anti-tumor growth effect was even superior to that of the positive control group, Ranolazine. Ki67 is a widely used marker for cell proliferation and has been proposed as a prognostic marker for cancer [28]. Immunofluorescence analysis of Ki67-positive (Ki67^+^) cells in breast tumor tissue (Fig. 4D, E) revealed that the percentage of Ki67-positive cells in the BmK-M9 treatment group was significantly lower than that in the control group. This suggests that BmK-M9 can effectively suppress the proliferation of tumor cells.

### mRNA Sequencing Analysis of the Mechanism of BmK-M9 in Breast Cancer

**Fig. 5:**
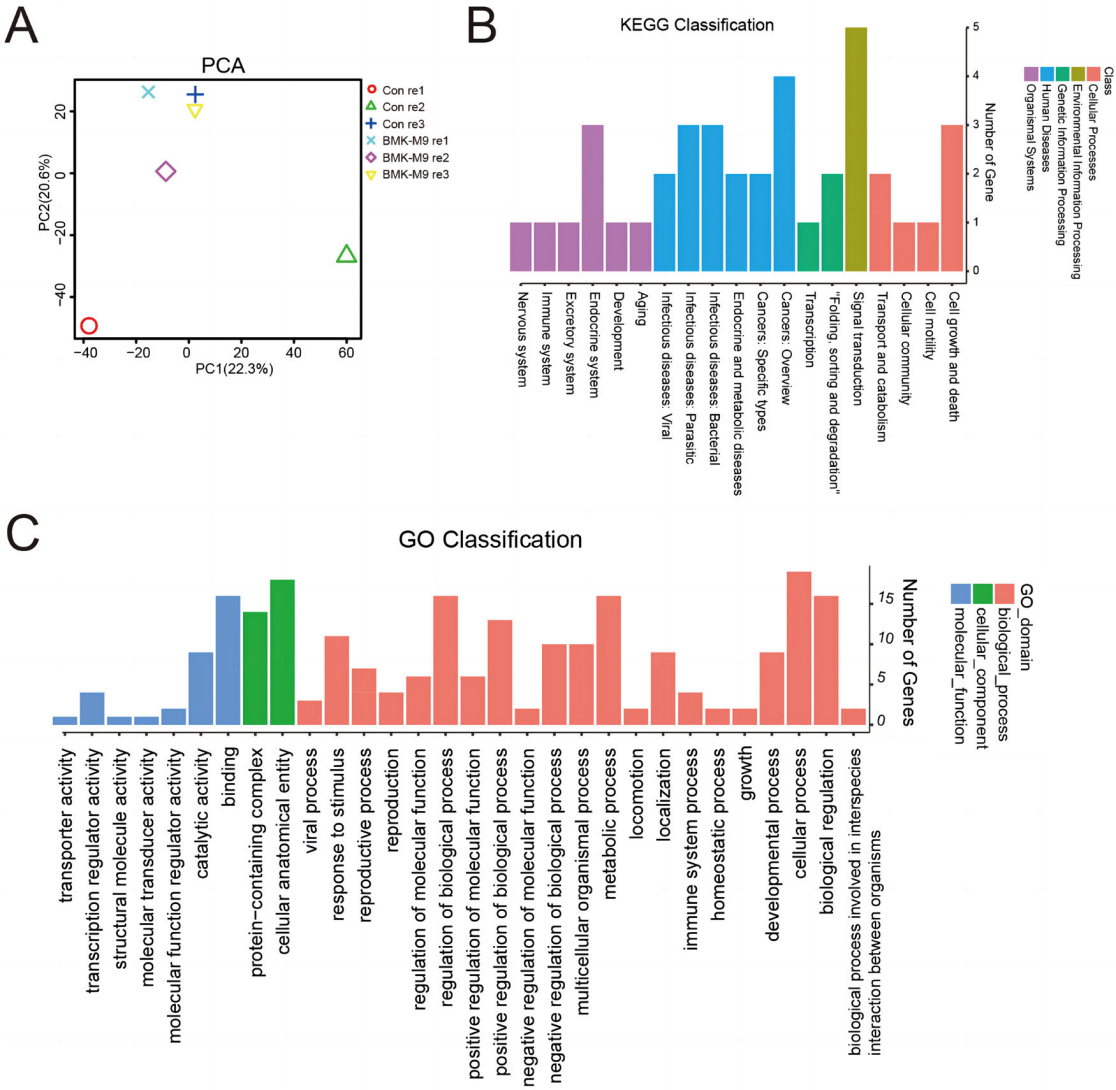
BMK-M9 mRNA Sequencing Analysis. **A** Pearson Correlation Analysis. **B** GO Class Analysis of Differentially Expressed Genes. **C** KEGG Class Analysis of Differentially Expressed Genes

To gain further insights into the anti-tumor mechanism of BmK-M9, MDA-MB-231 cells were treated with 1 μM BmK-M9 for 48 h. Subsequently, RNA was extracted from the cells using TRIZOL, and mRNA sequencing was performed. Principal Component Analysis (PCA) was conducted to assess the overall gene expression patterns, revealing no significant differences between the samples before and after BmK-M9 treatment (Fig. 5A). KEGG pathway enrichment analysis of the differentially expressed genes (DEGs) indicated enrichment in Cellular Processes, including cell growth and death, cell motility, cellular communication, transport, and catabolism (Fig. 5B). Similarly, Gene Ontology (GO) analysis showed a predominant enrichment in the biological process category, particularly related to the regulation of biological processes and growth (Fig. 5C).

### Functional Enrichment Analysis of BmK-M9 Effects on Tumor Cells using mRNA Sequencing

**Fig. 6:**
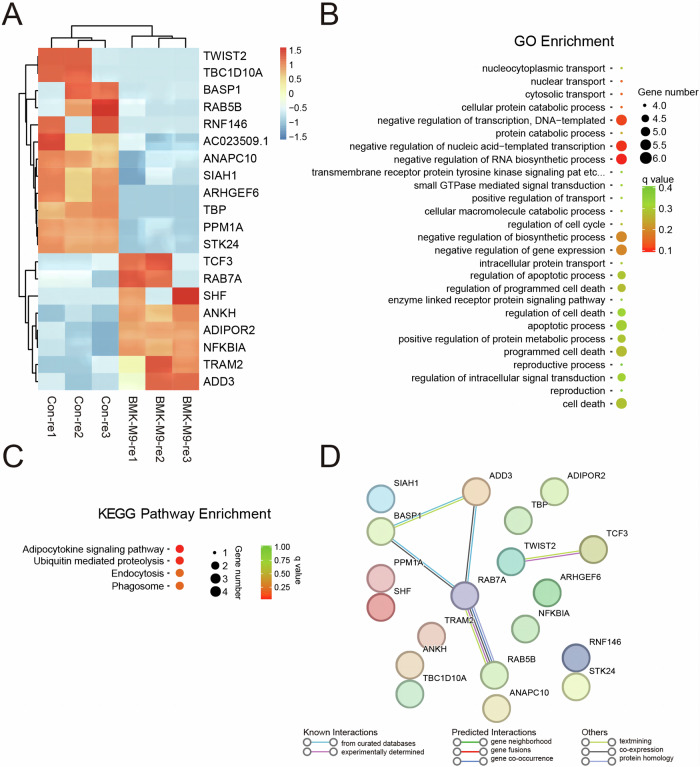
Functional Enrichment Analysis of mRNA Sequencing. **A** Heatmap representation of differentially expressed genes. **B** Gene Ontology (GO) functional enrichment analysis of biological processes. **C** KEGG pathways associated with tumorigenesis. **D** Protein-protein interaction network analysis using the STRING database

The gene expression profile analysis identified significant changes in 20 genes, with 12 genes downregulated and 8 genes upregulated (Fig. 6A). Subsequent Gene Ontology (GO) enrichment analysis revealed that these genes were primarily enriched in negative regulation of RNA transcription, including negative regulation of transcription, DNA-templated, negative regulation of nucleic acid-templated transcription, and negative regulation of RNA biosynthetic process (Fig. 6B). Notably, KEGG pathway analysis identified several intriguing pathways associated with the differentially expressed genes. These pathways include the adipocytokine signaling pathway (involving ADIPOR2 and NFKBIA), ubiquitin-mediated proteolysis (involving ANAPC10 and SIAH1), endocytosis (involving RAB5B and RAB7A), and phagosome (also involving RAB5B and RAB7A) (Fig. 6C). Furthermore, protein-protein interaction analysis using STRING revealed interactions between BASP1, ADD3, RAB7A, and RAB5B among the differentially expressed genes. Additionally, RAB7A and RAB5B were found to be enriched in the endocytosis and phagosome pathways (Fig. 6D).

### BmK-M9’s Impact on the β-catenin Signaling Pathway In Vitro and In Vivo

**Fig. 7:**
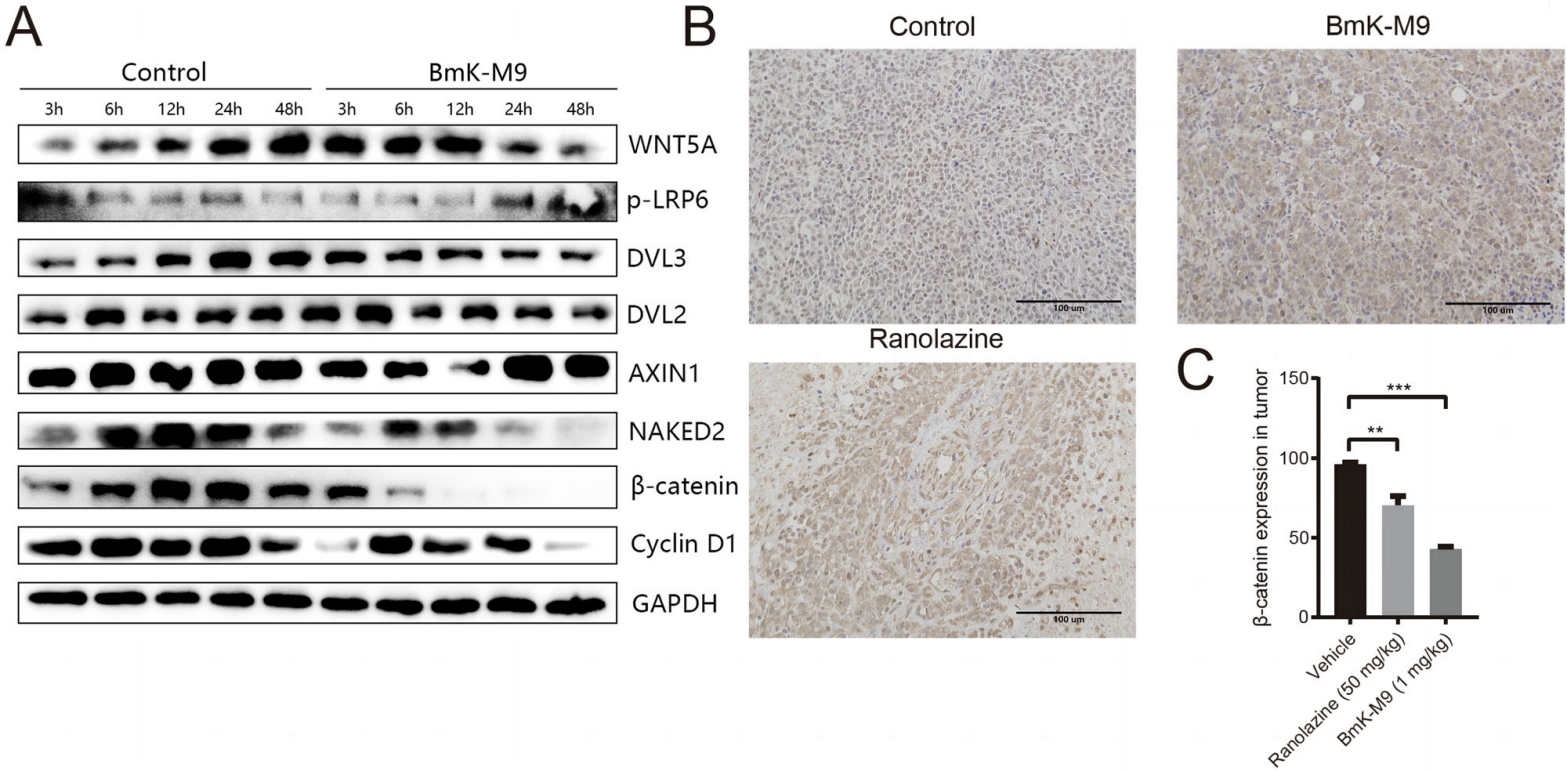
BmK-M9 affects the β-catenin signaling pathway in vitro and in vivo. **A** Western blot analysis of β-catenin signaling pathway-related proteins at 3, 6, 12, and 24 h after treatment with BmK-M9. **B** Immunohistochemical detection of β-catenin expression in tumor tissues from different groups of mice treated with BmK-M9. **C** Statistical analysis of β-catenin expression levels shown in Fig. B

To investigate whether BmK-M9 affects the β-catenin signaling pathway in the treatment of breast cancer, the expression of proteins related to the β-catenin signaling pathway was examined in MDA-MB-231 cells after treatment with BmK-M9 (Fig. 7A). The results revealed that the expression of key proteins associated with the β-catenin signaling pathway, such as DVL3, NAKED2, and β-catenin, consistently decreased in a time-dependent manner following BmK-M9 treatment in breast cancer cells. Furthermore, immunohistochemical analysis of mouse tumor tissue was employed to evaluate the expression of β-catenin. The results demonstrated that the expression of β-catenin in the BmK-M9-treated group was significantly reduced compared to the control group. Remarkably, the downregulation of β-catenin expression in the BmK-M9-treated group was even more pronounced than that observed in the positive control group (Fig. 7B, C). These findings suggest that the effects of BmK-M9 on breast cancer may be associated with the β-catenin signaling pathway.

## Discussion

Scorpion toxins, including peptides extracted from scorpion venom, have shown promise as potential candidates for anticancer therapy, including breast cancer [25, 29–31]. BmK-M9, a recombinant peptide derived from scorpion venom, has been demonstrated to inhibit the inactivation of sodium channels and exhibits properties of both α-toxins and β-toxins [12]. BmK-M9 exhibits highly efficient anticancer properties and can be expressed and purified through in vitro vectors, primarily playing a role in inhibiting tumor metastasis. Compared with other known anticancer peptides, BmK-M9 exerts its anticancer effects by inhibiting Na_V_1.5. Since the Na_V_1.5 channel is present in myocardial tissue, BmK-M9 may also have potential antiarrhythmic effects in patients with rapid arrhythmias. In this study, we synthesized BmK-M9 and verified its bioactivity on HEK293T cells overexpressing Na_V_1.5. BmK-M9 was found to significantly inhibit the invasion of MDA-MB-231, SUM149PT, and MCF-7 breast cancer cells. Moreover, we observed a significant inhibitory effect of BmK-M9 on tumor cell migration. Although the inhibition of cell viability and induction of apoptosis did not show a significant effect, there was a trend towards enhanced inhibition with increasing concentration of BmK-M9. Notably, our animal studies demonstrated a significant tumor-suppressive effect of BmK-M9.

The nude mouse model provides a physiologically relevant system for evaluating the anticancer activity of BmK-M9 in vivo. Notably, the formation of tumor-associated blood vessels facilitates effective drug delivery, which may explain the superior efficacy observed in in vivo experiments compared to in vitro studies. Several key factors contribute to the reliability of in vivo models for assessing anticancer drug activity. First, the complexity of the tumor microenvironment—including the extracellular matrix, diverse cell populations, and intricate signaling pathways—plays a critical role in tumor growth and progression [32–34]. Second, drug metabolism and pharmacokinetics influence therapeutic efficacy by determining how drugs are absorbed, distributed, metabolized, and excreted within the body. Additionally, the immune system is a crucial determinant, as immune cells within the tumor microenvironment can either enhance or suppress the antitumor response [35]. Finally, tumor heterogeneity and clonal evolution pose significant challenges, as tumors consist of diverse subpopulations with distinct genetic and phenotypic characteristics, leading to variable drug responses [36–38].

A previous study demonstrated that Na_V_1.5 increases intracellular sodium ion concentration, leading to the activation of the sodium-hydrogen exchanger 1 (NHE1) [39]. This activation results in intracellular alkalinization and extracellular acidification, thereby promoting cancer cell invasion and migration. As an Na_V_1.5 inhibitor, BmK-M9 exerts its effects by suppressing sodium channel activity, thereby inhibiting the invasive potential of breast cancer cells.

Additionally, BmK-M9 has been shown to regulate the Wnt/β-catenin signaling pathway, which plays a pivotal role in epithelial-mesenchymal transition (EMT) — a fundamental process driving cell invasion and migration. While the Wnt/β-catenin pathway also influences cell proliferation and apoptosis, these processes are regulated by multiple signaling pathways. Therefore, inhibition of Wnt/β-catenin signaling may be partially compensated by alternative pathways.

The concentration of sodium ions ([Na^+^]) has been closely associated with the invasion and metastasis of breast cancer cells, and elevated tumor [Na^+^] has been detected using Na-magnetic resonance imaging (MRI) in breast cancer [40]. BmK AGAP, a sodium channel inhibitor, has been shown to have analgesic effects through down-regulation of PTX3 via the NF-κB and Wnt/β-catenin signaling pathway [25, 41]. Kampo et al. observed that reduced secretion of β-catenin, both in vitro and in vivo, led to a decrease in breast cancer cell stemness and epithelial-mesenchymal transition. In our study, we observed that BmK-M9 was able to reduce β-catenin expression in MDA-MB-231 cells and solid tumors, resulting in tumor suppression.

Overexpression of TWIAT2 has been shown to activate the epithelial-to-mesenchymal transition program and enhance self-renewal of cancer stem-like cells, thereby promoting breast cancer progression [42, 43]. The upstream regulator CD44 exhibits a positive correlation with Na^+^/H^+^ exchanger isoform 1 (NHE1) [44]. RAB7A is highly expressed in breast cancer tissues and promotes cancer cell proliferation and invasion. Conversely, knockdown of RAB5B inhibits breast cancer cell proliferation, migration, and invasion [45]. The antagonistic relationship between RAB7A and RAB5B may represent a defensive mechanism employed by tumor cells to counteract the inhibitory effects of BmK-M9, which could disrupt vesicular transport processes critical for tumorigenesis. This disruption may consequently inhibit the nuclear translocation of β-catenin [46–48]. These findings suggest that BmK-M9 may not only inhibit the Wnt/β-catenin signaling pathway through direct downregulation of β-catenin but may also exert its effects by interfering with key regulatory factors involved in intracellular transport.

ANAPC10 has been relatively less studied in breast cancer, but its overexpression has been reported in non-small cell lung cancer cell lines. Knockdown of ANAPC10 has been shown to downregulate glutamine metabolism-induced autophagy and effectively inhibit proliferation and migration of non-small cell lung cancer cells [49, 50]. Adiponectin has been found to directly regulate the growth of normal breast epithelial cells and breast cancer cells through the ADIPOR1 and ADIPOR2 receptors [51].

The NIK/NF-kappaB pathway downregulates PTEN, leading to activation of the PI3K/Akt pathway. SIAH1, a critical E3 ubiquitin ligase, has been reported to mediate the degradation of β-catenin [52]. Our sequencing data reveal a downregulation trend in SIAH1 expression, which appears to contradict its inhibitory effect on β-catenin. However, previous studies indicate that downregulation of SIAH1 may impair the ability of tumor cells to adapt to stress conditions, thereby indirectly enhancing their sensitivity to apoptosis and inhibiting migration [53, 54]. Given that SIAH1 is involved in the Wnt/β-catenin signaling pathway, its regulation by BmK-M9 may represent an additional layer of regulatory complexity. Moreover, we observed a downregulation of RAB5B and upregulation of RAB7A, two small GTPases closely linked to intracellular transport and tumor progression. The antagonistic relationship between these proteins suggests that the upregulation of RAB7A could serve as a compensatory mechanism for the inhibition of Wnt/β-catenin, a hypothesis that warrants further investigation.

**Fig. 8:**
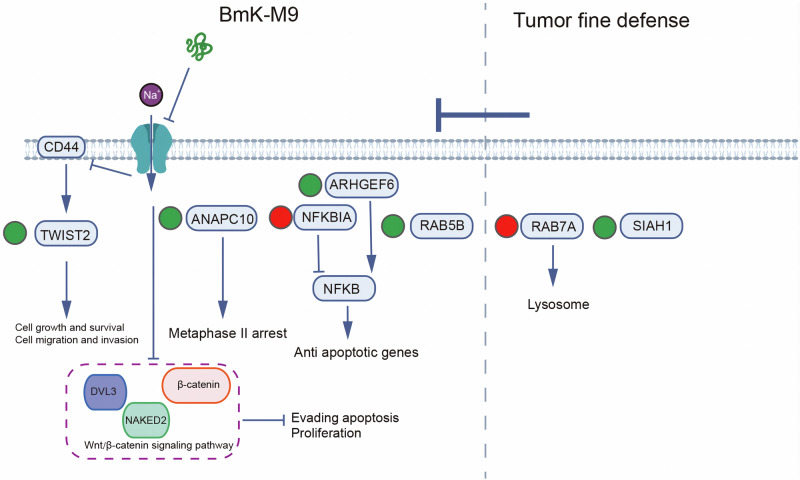
Schematic representation of the molecular mechanism underlying the inhibitory effects of BmK-M9 on breast cancer

Therefore, we examined genes related to the Wnt pathway and found downregulation of DVL3, NAKED2, and β-catenin. In this study, we examined genes associated with the Wnt pathway and observed a downregulation of DVL3, NAKED2, and β-catenin. These findings suggest that BmK-M9 primarily exerts its anticancer effects by inhibiting the Wnt/β-catenin signaling pathway, which is well-established for its role in promoting tumor invasion and metastasis [55, 56]. Notably, DVL3 and NAKED2 are crucial upstream regulators of β-catenin stability and transcriptional activity, and their inhibition further corroborates the suppressive effect of BmK-M9 on the Wnt/β-catenin pathway [57, 58]. These findings reinforce the mechanistic link between BmK-M9 and Wnt/β-catenin signaling suppression, highlighting its potential as a candidate for targeted anticancer therapy. Additionally, BmK AGAP, a Na(v) channel inhibitor commonly used as an analgesic, has been shown to downregulate PTX3 via the NF-κB and Wnt/β-catenin pathways. The upregulation of SIAH1, similar to RAB5B, may function as a defense mechanism in tumor cells. Therefore, when considering AGAP as a potential therapeutic for tumors, the concurrent use of SIAH1 inhibitors should be considered to mitigate the development of tumor resistance (Fig. 8).

## Conclusion

In conclusion, our study provides evidence that BmK-M9 exhibits anti-breast cancer effects both in vitro and in vivo. The underlying mechanism involves the inhibition of β-catenin expression, leading to the suppression of the Wnt/β-catenin signaling pathway and subsequent inhibition of tumor development.

## Data Availability

All study data are included in the article. The original data of RNA sequencing is available at NCBI SRA under the accession number.
